# Assessment of the variability of the morphological traits and differentiation of *Cucurbita moschata* in Cote d’Ivoire

**DOI:** 10.1038/s41598-023-30295-7

**Published:** 2023-03-06

**Authors:** Dagou Seka, Badié Arnaud Kouago, Beket Severin Bonny

**Affiliations:** School of Natural Sciences, University Nangui Abrogoua, 02 BP 801 Abidjan 02, Abidjan, Côte d’Ivoire

**Keywords:** Ecology, Evolution, Genetics, Plant sciences

## Abstract

With its predisposition to adapt to different environments, *Cucurbita moschata* grows well in various ecosystems. It is not a very exigent plant and has an inherent capacity for plasticity that underlies its large variability. An assessment of the accessions of *C. moschata* in Cote d’Ivoire shows that the plant exhibits large morphological and phenological variability for all the 28 measured traits. There are outliers among most measured traits. Further analysis indicates the emergence of three ecotypes in congruence with the three distinct ecosystems and their respective bioclimatic characteristics. In the savannah region, characterized by a short rainy season followed by a long dry season, a total yearly rainfall of 900 mm, an elevated daily temperature of 29 °C, and a high relative humidity of 80%, the cline of *C. moschata* is long and thin, with small leaves, small peduncles and small fruits. It has a high growth rate and accelerated phenology. The mountain region has a very long rainy season followed by a short dry season, a total pluviometry of 1400 mm, an average daily temperature of 27 °C and a relative humidity of 69%. The cline of *C. moschata* in the mountain region is characterized by a delayed flowering and a delayed fruit maturity, large number of small seeds and large fruits. The forest region has a favorable climate for the growth of *C. moschata* in Cote d’Ivoire. It has two rainy seasons that alternate with two dry seasons of unequal durations, an annual rainfall of 1200 mm, an average daily temperature of 27 °C and a relative humidity of 70%. The cline of *C. moschata* in that region has a large girth size, large dimensions of the leaves, long peduncles and larger and heavier fruits. The seeds are also large but in small number. It appears that the anatomy and physiology of the clines are differentiated primarily in response to soil water content and availability for the ontogeny of the plant.

## Introduction

The migration of species in a new environment means that the environment offers favorable conditions for the species’ survival and accommodation. Such migration and accommodation are often accompanied by the expression of different phenotypes depending on the environment and the interaction between genotype and environment^1–6^. Their persistence in the new environment along with natural selection result in their adaptation, and other time, increase their fit to the environment^7–9^. The processes leading to the fitness of the species in the new environment involve natural random mating and genes transfer from parents to progeny^10^, mutation and genes recombination which result in an assortment of genotypes with dominance, co-dominance, and recessive individuals. Natural selection eventually removes the purely recessive genotypes from the population as they often cannot survive in the new environment. These processes occur gradually and lead to genetic variability. An illustration is the different degrees of resistance to pesticides or heavy metal toxins^11,12^.

Genetic variability is the driving force of species persistence and evolution. It is the natural insurance policy as it provides an insurance for the species survival against the unexpected. Natural random mating allows the transfer of genes from individuals to others. In addition to the gene transfer, the recombination of genes and genetic mutation are the main cause of genetic variability that insures the survival and the evolution of species. In agricultural research, genetic variability is the backbone of cultivar improvement^13–19^. It makes the genetic resources available for undertaking the breeding program for crop improvement. For example, the introgression of quantitative trait loci on southern leaf blight resistance in maize hybrids^20^ was feasible due to the genetic variation for the disease. Modern biotechnology tools can be used to introduce foreign genes into the genome of an individual^21^, thereby enhancing the genetic variability of the species as the introduced genes are heritable and can be passed to the progeny. With the importance of genetic variability in a breeding program, germplasm collection, maintenance and regular assessment have been an important part of the activities in research institutions^22–24^. The current study concerns the *Cucurbita moschata* germplasm in Cote d’Ivoire, precisely its morphological characterization as part of a program of *Cucurbita moschata* improvement.

*Cucurbita moschata* originated from Latin America^25,26^ and was an important crop for the indigenous people who first cultivated the crop for its edible seed and fruit^27^. From Latin America where it is first cultivated, *Cucurbita moschata* migrated to the Caribbean Islands where it adapted to the local ecosystems and further diversified^28,29^. After the discovery of America and through the intercontinental voyages that followed, *Cucurbita moschata* was spread to Europe, Asia and Africa and became naturalized to those continents, with increased genetic diversity in the new environments^27,30^. The diversification that resulted from the adaptation and acclimation of *C. moschata* to the diverse regions of the world created geographical subspecies^31^. Other studies add support to the geographical subspecies formation with the identification of distinct clusters based on geographic origins^19,32,33^.

*C. moschata* is an annual crop that grows well in warm tropical areas^17,34–36^. It is even called “tropical pumpkin”^35^. It is very well suited to the climatic conditions of Africa. Unfortunately, it is not yet an important revenue-generating crop in Africa and it is neglected across the whole continent. This neglect is especially true in Cote d’Ivoire. Cote d’Ivoire is a country where agriculture occupies more than 60% of the active population and contributes up to 35% to its gross domestic product. However, most of the agricultural research activities and crop promotion are focused on cash crops for export. Food crops for local consumption have received very little attention from the public authorities. And *C. moschata* has totally been ignored in agricultural research programs until now. In Cote d’Ivoire, *C moschata* is sparsely cultivated by small-scale farmers. To date, they are the only ones with any information on the vegetable crop. Given its lauded nutritional and medicinal importance^34^, consumption of the fruit pulp, the seed, the leaves and the flowers in diverse dishes is growing in Cote d’Ivoire and necessitates programs for the collections of the various accessions of *C. moschata*, the characterization, the conservation and the improvement of *C. moschata* genetic resources in Cote d’Ivoire. We conducted the present study to determine the agro-morphological characteristics of the different accessions of *C. moschata* collected from the main growing regions of Cote d’Ivoire and a region in Burkina Fasso. Because the environmental conditions of the growing regions are strikingly different, we evaluated the possibility of ecotypes of *C. moschata* in relation to those ecosystems. All the inferences in this study are based on the quantitative measures of the vegetative, phenological and yield traits.

## Results

### Description of the phenological, vegetative and yield traits of the accessions per habitat

The process of data management included the computation of mean squares for the assessed phenological, vegetative and yield traits of the accessions with the sampling habitats considered as the treatment factor. The error mean squares served in the multiple comparison of means reported in Table 1.

**Table 1:** Means of the measured phonological, vegetative and flowering and yield traits of *Cucurbita moschata* genotypes sampled from seven habitats.

Traits	Tiassale	Soubre	Zh	Korho	Ferke	Bondu	Burki
Phenological							
DTE	5.889ab	5.789ab	5.162bc	6.067a	4.984c	5.382b	4.027d
DTF	108.778a	95.526b	103.044a	74.840c	75.593c	69.450d	65.333e
DFM	100.444a	96.526a	102.394a	72.933bc	73.562b	69.362c	62.819d
DFF	107.222a	103.421a	108.143a	96.360b	92.140c	87.281d	68.638e
DPM	152.889a	151.526a	153.950a	137.506b	137.984b	137.656b	114.042c
AFM	44.111bc	47.315ab	42.384bc	41.284bc	46.656b	53.04a	40.00c
Vegetative							
PLH	471.80bc	456.42bc	417.38c	423.98bc	489.20b	586.91a	446.16bc
GSF	4.43ab	4.63a	3.83c	3.80c	3.90bc	3.93bc	3.96bc
LOL	24.98a	19.27b	16.57c	14.96d	15.79cd	15.96c	15.61cd
WOL	19.94a	16.91b	14.70c	13.35d	13.89d	13.68d	14.29cd
MPL	16.26a	10.65b	6.87d	7.51d	7.44d	9.03c	7.49d
FPL	4.03a	2.49ab	2.39ab	3.50a	2.52ab	2.87ab	2.44ab
LOP	34.94a	26.21b	21.15e	22.89cd	22.10de	24.70b	23.74bc
Flowering and Yield							
NMF	27.33a	22.58ab	10.85d	16.52c	15.33c	19.72b	11.35d
NFF	5.22a	6.05a	2.30b	1.93bc	1.57c	2.14b	1.92bc
NFP	2.78a	2.53a	1.49b	1.45b	1.36bc	1.24c	1.31bc
LOF	40.87a	38.19a	21.48d	22.07d	22.12d	24.38c	29.05b
DOF	27.37a	23.37b	15.99c	11.17f	12.45e	11.79ef	14.16d
VOF	11,155.80a	8506.30b	2499.20c	932.50e	1218.90de	1397.00de	1712.30d
WOF	7288.86a	5856.84b	2231.38c	1170.78e	1438.23de	1579.17d	2438.05c
DCE	19.02a	15.31a	10.84b	6.46d	7.49cd	7.99c	8.48c
TTM	5.30a	4.91a	2.91c	3.01bc	3.286b	2.356d	3.07bc
WTM	6472.22a	6189.47a	1636.65b	882.53d	1100.86cd	1198.62c	1616.81b
NOS	162.22d	143.84d	359.64a	365.89a	288.78b	257.71c	169.89d
WFS	40.13cd	39.66d	59.26b	71.33a	50.90c	56.95b	34.16d
LDS	1.73a	1.59b	1.28e	1.51c	1.58b	1.38d	1.67a
WIS	0.95a	0.91a	0.80b	0.79b	0.81b	0.71c	0.89a
WDS	23.90cd	20.26d	34.83b	44.97a	32.22bc	32.81b	15.14d

Read horizontally to compare the means of the accessions from different habitats. Means followed by the same letter are not significantly different ($\alpha = 0.05$). Read vertically to assess the accessions of a habitat over all the measured traits.

Regarding the phenological traits, the accessions from the habitat of Zh have the longest period from seeding to first male (102.39 d) and first female (108.14 d) flower appearances, and the longest period from seeding to physiological maturity (153.95 d). For those traits, the accessions from Tiassale and Soubre are not significantly different from those of Zh. And, accessions from Tiassale and Zh have the longest periods from seeding to 50% flowering. On the other hand, accessions from Korho, Ferke, Bondu and Burki develop their first male and female flowers and attain 50% flowering in a very short period. They also reach physiological maturity faster. Accessions from Korho, however, have the longest period from seeding to 50% emergence (6.07 d) and accessions from Bondu have the longest period from first female flower appearance to physiological maturity (53.04 d).

For the vegetative traits, accessions from Tiassale and Soubre have the largest girth size (4.43 cm and 4.63 cm, respectively). Accessions from Tiassale have the longest (24.98 cm) and widest (19.94 cm) leaves, the longest male (16.2 cm) and female (4.03 cm) peduncles and the longest petioles (34.94 cm). The measures for those organs on accessions from Soubre rank second to those of Tiassale. On the other hand, accessions from Korho, Ferke, Bondu and Burki are characterized by smaller girth size, smaller leaves, smaller petioles and smaller peduncles of male and female flowers. But the accessions from Bondu are the tallest (586.91 cm) followed by the accessions from Ferke (489.20 cm). And the accessions from Zh are the shortest (417.38 cm).

For the flowering and yield traits, accessions from Tiassale and Soubre show the largest numbers of male (27.33 units and 22.58 units, respectively) and female (5.22 units and 6.05 units, respectively) flowers per plant, largest numbers of fruits per plant (2.78 units and 2.53 units, respectively) and largest measures of all fruit-related traits. Their seeds are very large, but in small numbers. In contrast, accessions from Korho, Ferke, Bondu and Burki have the smallest numbers of male and female flowers per plant, the smallest numbers of fruits per plant and the smallest measures of fruit-related traits. They have large numbers of seeds, but their seeds are smaller, except the seeds of the accessions from Burki. Refer to Table 1 for more detailed information.

### Variability of the phenological, vegetative and yield traits

Table 2 shows the spread of the phenological and morphological traits of the assessed accessions of *C. moschata*. All the evaluated traits showed very wide ranges of distribution of the observations. Some conspicuously wide ranges of traits include number of days to 50% flowering (DTF) that goes from 52 to 152 d, plant height with a minimum of 48 cm and a maximum of 1510 cm, diameter of the fruit that is between 5.8 cm and 35 cm, weight of the fruit that varies between 150 g and 10,930 g and number of seeds per fruit that spreads in the interval from 32 units per fruit to 729 units per fruit. Excluding the number of days to 50% emergence (DTE), all the other assessed traits have remarkably wide ranges of phenotypic expressions (Table 2). All the traits but DTE, DTF, days from first female flower appearance to fruit maturity, fruit length and length of the dry seed, had outliers. The number of outliers ranged from 1 to 67. Except the outliers observed with the width of the dry seed, all the outliers were above 1.5*IQR + Q3 where IQR is the inter-quartile range and Q3 is the third quartile. The presence of outliers is indicative of the richness and large variability of the population of accessions. The outliers are exceptional performances that fall outside the normal distribution of the observations. They are a stock of unusual traits that can be used in a crop improvement program when beneficial. For example, the observed outliers for diameter of the fruit, weight of the fruit or thickness of the pulp can be used in a breeding program for the improvement of fruit yield. Similarly, outliers for beneficial traits related to the seed can be used to improve *C. moschata* crop for seed yield. Besides, the computed mean squares (data not reported) showed highly significant variations between accessions for the assessed traits. They all yielded p-values less than 0.01, providing additional support to the evidence of large variability among the accessions of *C. moschata* of Cote d’Ivoire. The computed standard deviation, and median absolute deviation for each trait are additional evidence. We should note that in most cases, the mean squares associated to year (data not reported) were not significant, indicating the relative stability of the assessed traits.

**Table 2:** Minimum (Min), first quartile (Q1), median, third quartile (Q3), maximum (Max), standard deviation (SD), median absolute deviation (MAD) and outliers obtained from the phenological, vegetative and flowering and yield traits of 663 accessions of *C. moschata*.

Traits	Min	Q1	Median	Q3	Max	SD	MAD	Outliers
Phenological								
DTE	4.00	4.00	5.00	6.00	9.00	1.31	0.98	0
DTF	52.00	65.00	79.00	98.00	152.00	19.95	16.96	0
DFM	52.00	62.00	77.00	97.00	151.00	21.03	17.96	2
DFF	52.00	4.00	93.00	106.00	157.00	18.42	14.45	4
DPM	87	126	143	153	205	20.25	16.17	3
AFM	16.00	36.00	48.00	56.00	70.00	13.41	11.09	0
Vegetative								
PLH	48.00	320.00	425.00	593.00	1510.00	240.70	179.19	19
GSF	2.40	3.40	3.80	4.20	8.80	0.89	0.58	38
LOL	8.90	13.50	15.60	18.30	31.80	3.89	2.97	16
WOL	7.90	11.90	13.70	15.80	26.70	3.26	2.51	26
MPL	2.30	5.50	7.40	9.50	28.00	3.26	2.39	19
FPL	1.00	1.85	2.50	3.10	8.00	1.02	0.79	15
LOP	12.00	19.00	21.60	25.30	48.40	6.09	4.40	39
Flowering and yield								
NMF	1.00	10.00	14.00	18.00	70.00	7.62	0.01	29
NFF	1.00	1.00	2.00	3.00	24.00	1.74	5.76	8
NFP	1.00	1.00	1.00	2.00	5.00	0.73	0.42	11
LOF	9.60	16.90	22.30	29.00	46.60	8.29	6.87	0
DOF	5.80	10.20	13.40	16.10	35.00	5.04	3.80	26
VOF	55.37	555.65	1287.60	2185.12	22,449.30	2579.68	1398.91	67
WOF	150.00	1000.00	1600.00	2400.00	10,930.00	1576.82	1033.56	50
DCE	2.00	5.90	9.00	10.30	27.80	3.56	2.69	13
TTM	0.70	2.20	3.00	3.45	7.20	1.11	0.81	35
WTM	80.00	645.00	1140.00	1840.00	10,600.00	1467.14	895.37	52
NOS	32.00	189.00	296.00	390.00	729.00	136.17	111.79	1
WFS	6.45	38.62	55.70	68.04	140.32	23.15	18.15	12
LDS	0.80	1.28	1.43	1.60	2.00	0.22	0.18	0
WIS	0.30	0.70	0.80	0.90	1.15	0.13	0.10	4
WDS	3.01	20.99	33.52	43.33	76.85	15.06	12.24	1

The components of variance, the quantitative genetic differentiation, the overall mean, and the coefficients of variation are reported in Table 3. The *lme4* package^37^ used in the determination of the components of variance, does not provide p-values in the analysis of mixed or random models. The reported quantities in Table 3 are not accompanied with tests of significance. It is worth mentioning that the respective units of measure of the assessed traits are squared for the variances and the evaluated estimates will be reported without the units of measure. The phenotypic variance ($\sigma_{p}^{2}$) is partitioned into variance between morphotypes or genotypic variance ($\sigma_{g}^{2}$), and within morphotypes or residual variance ($\sigma_{e}^{2}$). For the class of phenological traits, considerable genotypic variances were observed with days to 50% flowering (266.21) and days to first male flower appearance (254.40), compared with their respective residual variances (148.13 and 199.50). Regarding the class of vegetative traits, only the peduncle length of male flowers had a genotypic variance (9.22) greater than its residual variance (8.86). In the class of flowering and yield traits, 8 of the 15 traits assessed showed large genotypic variances in comparison with their respective residual variances. They are number of female flowers per plant ($\sigma_{g}^{2}$ = 3.02 versus $\sigma_{e}^{2}$ = 2.36), length of the fruit ($\sigma_{g}^{2}$ = 53.96 versus $\sigma_{e}^{2}$ = 48.97), diameter of the fruit ($\sigma_{g}^{2}$ = 37.17 versus $\sigma_{e}^{2}$ = 16.76), volume of the fruit ($\sigma_{g}^{2}$ = 10,713,468 versus $\sigma_{e}^{2}$ = 3,904,590), weight of the fruit ($\sigma_{g}^{2}$ = 5,413,819 versus $\sigma_{e}^{2}$ = 1,420,187), diameter of the cavity enclosing the seed ($\sigma_{g}^{2}$ = 19.12 versus $\sigma_{e}^{2}$ = 7.75), thickness of the fruit pulp ($\sigma_{g}^{2}$ = 1.11 versus $\sigma_{e}^{2}$ = 0.94) and weight of the fruit pulp ($\sigma_{g}^{2}$ = 5,979,212 versus $\sigma_{e}^{2}$ = 1,088,750). For a trait to have a lager genotypic variance than the residual variance is synonymous to a relative ease of improvement of the crop for that trait through a breeding program.

**Table 3:** Components of variances ($\sigma_{p}^{2}$, $\sigma_{g}^{2}$, $\sigma_{e}^{2}$, $\sigma_{a}^{2}$), quantitative genetic differentiation ($Q_{ST}$), overall mean ($\mu$), and coefficients of variation (%) ($CV_{p}$,$CV_{g}$,$CV_{e}$), of the measured phenological, vegetative and yield traits of the accessions of *C. moschata* of Cote d’Ivoire.

Traits	$\sigma_{p}^{2}$	$\sigma_{g}^{2}$	$\sigma_{e}^{2}$	$\sigma_{a}^{2}$	$Q_{ST}$	$\mu$	$CV_{p}$	$CV_{g}$	$CV_{e}$
Phenological									
DTE	1.86	0.44	1.42	0.07	0.14	5.28	25.82	12.54	22.58
DTF	414.34	266.21	148.13	36.47	0.47	83.94	24.25	19.44	14.50
DFM	453.9	254.40	199.50	14.40	0.39	82.04	25.97	19.44	17.22
DFF	373.28	183.01	190.27	6.56	0.32	94.34	20.48	14.34	14.62
DPM	444.41	218.12	226.29	87.14	0.32	141.33	14.91	10.45	10.64
AFM	178.33	19.03	159.30	1.30	0.27	45.06	29.63	10.00	28.27
Vegetative									
PLH	57,273	3534	53,739	Na	0.03	477.62	50.10	12.45	48.54
GSF	0.78	0.03	0.75	0.07	0.02	3.97	22.24	4.36	21.90
LOL	21.79	8.81	12.98	1.33	0.25	17.35	26.90	17.11	20.76
WOL	13.00	3.57	9.43	0.79	0.16	15.021	24.00	12.58	20.45
MPL	18.07	9.22	8.86	0.15	0.34	9.20	46.20	33.01	32.35
FPL	6.68	0.12	6.56	0.02	0.01	2.75	93.98	12.60	93.12
LOP	45.28	13.32	31.96	3.06	0.17	24.68	27.26	14.79	22.90
Flowering and yield									
NMF	72.96	30.83	42.13	0.641	0.27	17.40	38.49	31.90	37.30
NFF	5.38	3.02	2.36	0.050	0.39	2.96	65.81	58.63	51.79
NFP	0.82	0.36	0.46	Na	0.28	1.43	50.13	41.84	47.87
LOF	102.93	53.96	48.97	9.84	0.36	27.80	31.09	26.09	29.28
DOF	53.92	37.17	16.76	0.13	0.53	16.50	39.62	36.94	24.80
VOF	14,618,058	10,713,468	3,904,590	Na	0.58	3887	89.17	84.20	50.83
WOF	6,834,006	5,413,819	1,420,187	98,667	0.66	3097.2	78.3	75.12	38.47
DCE	26.87	19.12	7.75	1.05	0.55	10.57	47.89	40.09	45.38
TTM	2.05	1.11	0.94	0.01	0.37	3.516	33.90	29.92	27.60
WTM	7,067,962	5,979,212	1,088,750	6224	0.73	2709.1	92.96	90.26	38.51
NOS	22,167.0	8231.00	13,936.00	Na	0.23	292.90	38.74	30.97	40.30
WFS	606.41	164.51	441.90	Na	0.16	54.93	32.148	23.351	38.27
WDS	270.93	98.74	172.19	Na	0.22	32.29	38.694	30.770	40.63
LDS	0.05	0.02	0.03	0.001	0.31	1.53	11.692	9.912	10.54
WIS	0.02	5.71 10^–3^	1.48 10^–2^	0.00005	0.16	0.83	17.26	9.10	14.70

The coefficient of variation (*CV*) is another statistic that measures variation. It is actually the dispersion of a trait per unit measure of its mean, which can be used to compare variations of traits with different measurement units or different scales. As a rule-of-thumb, a coefficient of variation greater than 20% is indicative of large variation for the trait. The phenotypic coefficient of variation is considerably high for 25 of the 28 assessed traits. Only the number of days from seeding to physiological maturity, the first and second longest axes of the dry seed show coefficients of variation less than 20%. Traits with very large phenotypic coefficients of variation include the peduncle length of female flowers ($CV_{p}$ = 93.98%), weight of the pulp ($CV_{p}$ = 92.96%), volume of the fruit ($CV_{p}$ = 89.17%), weight of the fruit ($CV_{p}$ = 78.30%) and number of female flowers per plant ($CV_{p}$ = 65.81%). With respect to the residual coefficients of variation, only the number of days from seeding to 50% emergence and number of days from first female flower appearance to physiological maturity have residual coefficients of variation greater than 20%, among the phenological traits. All the vegetative traits have residual coefficients of variation greater than 20%, and show a near-perfect linear relation (*r* = 0.98; *p* < 0.001) with the phenotypic coefficients of variation. From that observation, we may conclude that the variations in the phenotypic expressions of the vegetative traits are largely due to the variations within morphotypes. For the flowering and yield traits, all the residual coefficients of variation are greater than 20%, except the first and second longest axes of the seed. The genotypic coefficient of variation is less than 20% for all the phenological and vegetative traits except the peduncle length of male flowers with a coefficient of variation of 33.01%. On the other hand, 13 of the 15 flowering and yield traits have genotypic coefficient of variation greater than 20%. Among them, are the weight of the pulp with a genotypic coefficient of variation of 90.26%, the volume of the fruit with a genotypic coefficient of variation of 84.20% and the weight of the fruit with a genotypic coefficient of variation of 75.12%. Besides, for the flowering and yield traits, the genotypic coefficients of variation are highly correlated (*r* = 0.99, *p* < 0.001) with the phenotypic coefficients of variation in a near-perfect linear trend ($b = 0.97;p < 0.001$). That finding forms the basis to infer that most of the variations in the phenotypic expressions of the flowering and yield traits are largely caused by genotypic variability, without dismissing the contribution from the variability within morphotype.

The quantitative genetic differentiation, termed $Q_{ST}$^38^, is broadly the ratio of genotypic variance to phenotypic variance. It is closely related to the estimator of heritability. It scales between 0 and 1. It is well suited to the genetic analysis of morphological traits. In this study, the computed estimates of $Q_{ST}$ take values between 0.01 and 0.73. A value of $Q_{ST}$ = 0.28 is considered moderate quantitative genetic differentiation^38^. And it is easy to see that a value of $Q_{ST} = \tfrac{1}{3}$ implies that the between-morphotype variance is equal to the within-morphotype variance for a morphological or phenological trait. And a $Q_{ST}$ = 0.5 means the between-morphotype variance is twice the within-morphotype variance and can be qualified as a considerably large estimate of genetic differentiation. Based on our estimates of genetic differentiation, we may affirm that moderate to considerably large differentiation has occurred for several phenological, vegetative and yield traits and the differentiation is particularly high for fruit-related traits such as diameter of the fruit ($Q_{ST}$ = 0.53), diameter of the cavity enclosing the seeds ($Q_{ST}$ = 0.55), volume of the fruit ($Q_{ST}$ = 0.58), weight of the fruit ($Q_{ST}$ = 0.66), and weight of the pulp ($Q_{ST}$ = 0.73). The observed morphological differences of the accessions in the plots of the experimental trials led to the attempt to regroup the morphotypes of *C. moschata* in clusters with unsupervised methods. The results are given in the section below.

### Segmentation of the accessions and identification of ecotypes

**Figure 1:**
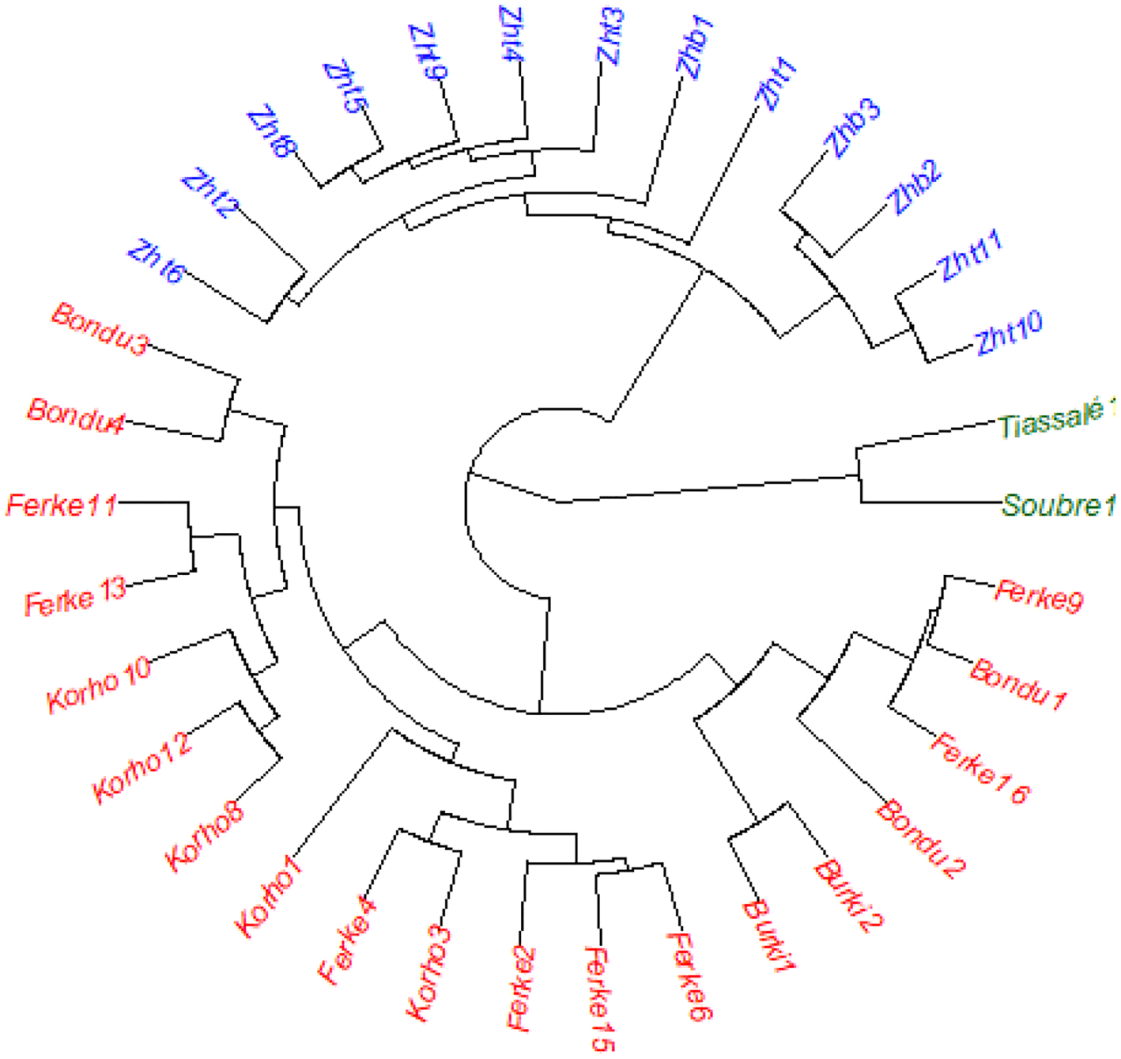
Circular phylogenetic tree of the 34 morphotypes of *C. moschata* grown in Cote d’Ivoire. The three colors define the grouping of the morphotypes according to their morphological and phenological similarities.

**Figure 2:**
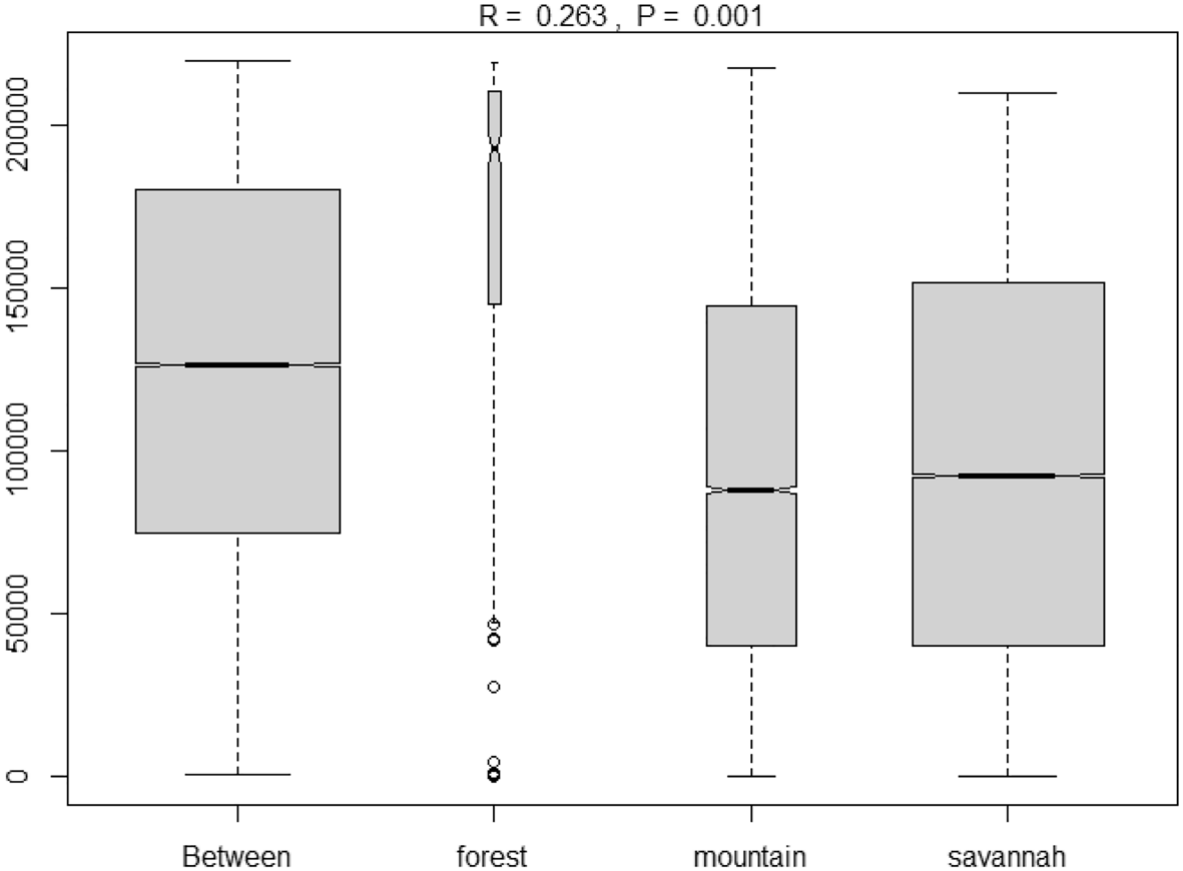
Rank order of dissimilarities between and within geographic regions of differentiation of the accessions of *C. moschata* of Cote d’Ivoire.

The clustering was first performed with base R^39^. The *NbClust* package^40^ determined 3 clusters based on the majority rule. The *hclust* object was then used with the *ape* package^41^ to create the circular phylogenetic tree of Fig. 1. The cluster validation was verified with the *fpc* package^42^. The tree regrouped morphotypes according to their phenological and morphological similarities in three clusters that also reflect the 3 geographical zones labeled forest, mountain, and savannah. The 3 zones are characterized by distinct bioclimatic parameters (seasons, rainfall, temperature and humidity, see Table 4). Accessions from the same geographical zone were similar and accessions from different zones were distant. In general the forest region is characterized by two rainy seasons that alternate with two dry seasons, an accumulated annual rainfall of 1200 mm, an average daily temperature of 27 °C and a relative humidity of 70%. The mountain region has one very long rainy season with an accumulated annual rainfall of 1400 mm and a short dry season, an average daily temperature of 27 °C and a relative humidity of 69%. The savannah region has a long dry season followed by a short rainy season with an accumulated annual rainfall of 900 mm, an elevated daily temperature of 29 °C, and an elevated relative humidity of 80%. The forest region includes the morphotypes of Tiassale and Soubre, the mountain region has the morphotypes of the habitat of Zh and the savannah region has the morphotypes of the habitats of Bondu, Ferke, Korho, and Burki. The K-means algorithm was also used to cluster the accessions and the results similarly showed that the accessions within a cluster were from the same geographical zone as defined above (data not shown). The three regions showed large genotypic diversity among the accessions of *C moschata*. Figure 2 gives a picture of the diversity of the accessions with the dissimilarity measures between and within geographic regions representing the main growing areas of *C. moschata*. The diversity is presented by the quartiles, the minimum and the maximum rank of the dissimilarities within a region. The width of the box is determined by the number of morphotypes considered in the drawing of the boxplot and is not related to the genotypic richness of the accessions in a region. The median dissimilarity within the forest region is ranked approximately 190000th with a total number of 220,000 dissimilarity points in the population. The forest region also presents some outliers which are accessions of *C. moschata* that are morphologically or phenologically distinct from the commonly observed morphology and phenology of *C. moschata* in Cote d’Ivoire. The forest region has the largest genotypic diversity. The genotypic diversity in the other two regions is also considerably large with median dissimilarity ranking about 85000th and 90000th, respectively for the mountain and the savannah regions. The minimum and maximum dissimilarity ranks of the mountain region are about the same as the minimum and the maximum dissimilarity ranks of the distribution of accessions between regions.

**Table 4:** Bioclimatic parameters, origin and distribution of the accessions of *Cucurbita moschata* assessed in this study.

Parameters	Habitats						
	Côte d’Ivoire						Burkina Faso
	Korho	Ferke	Bondu	Tiassalé	Soubré	Zh	Burki
Geographic coordinates	9°27′41’’ N–5°38′19’’ W	9°35′37’’ N–5°11′50’’ W	8°02′23’’ N–2°47′54’’ W	5°54’ N–4°50’ W	5°47′08’’ N–6°36′30’’ W	6°55’ N–8°13’ W	10°20’ N–3°11’ W
Annual rainfall	951.4 mm	940 mm	850 mm	1226 mm	1169 mm	1400 mm	727 mm
Temperature	29 °C	28,2 °C	28,4 °C	27 °C	27 °C	27 °C	29,1 °C
Relative humidity	88%	81%	87%	70%	70%	69%	77%
Seasons	2 seasons: Dry season: October-May Rainy season: June–September	2 seasons: Dry season: October-May Rainy season: June–September	2 Seasons: Dry season: October–April Rainy Season: May–September	4 Seasons: Long rainy season: April-July Short dry season: August–September Short rainy season: October–November Long dry season: December-March	4 Seasons: Long rainy season: March-June Short dry season: July–August Short rainy season: September–October Long dry season: November-February	2 seasons: Dry season: December- January Rainy season: February- November	2 seasons: Dry season: October-May Rainy season: May–September
Vegetation	Wooded savannah	Wooded savannah	Wooded savannah interspaced with gallery forest	Dense forest	Dense forest sometimes sparse	Dense forest	Wooded savannah
Number of accessions and (morpho-types) per habitat	11 (5)	10 (8)	19 (4)	5 (1)	5 (1)	25 (13)	10 (2)

**Figure 3:**
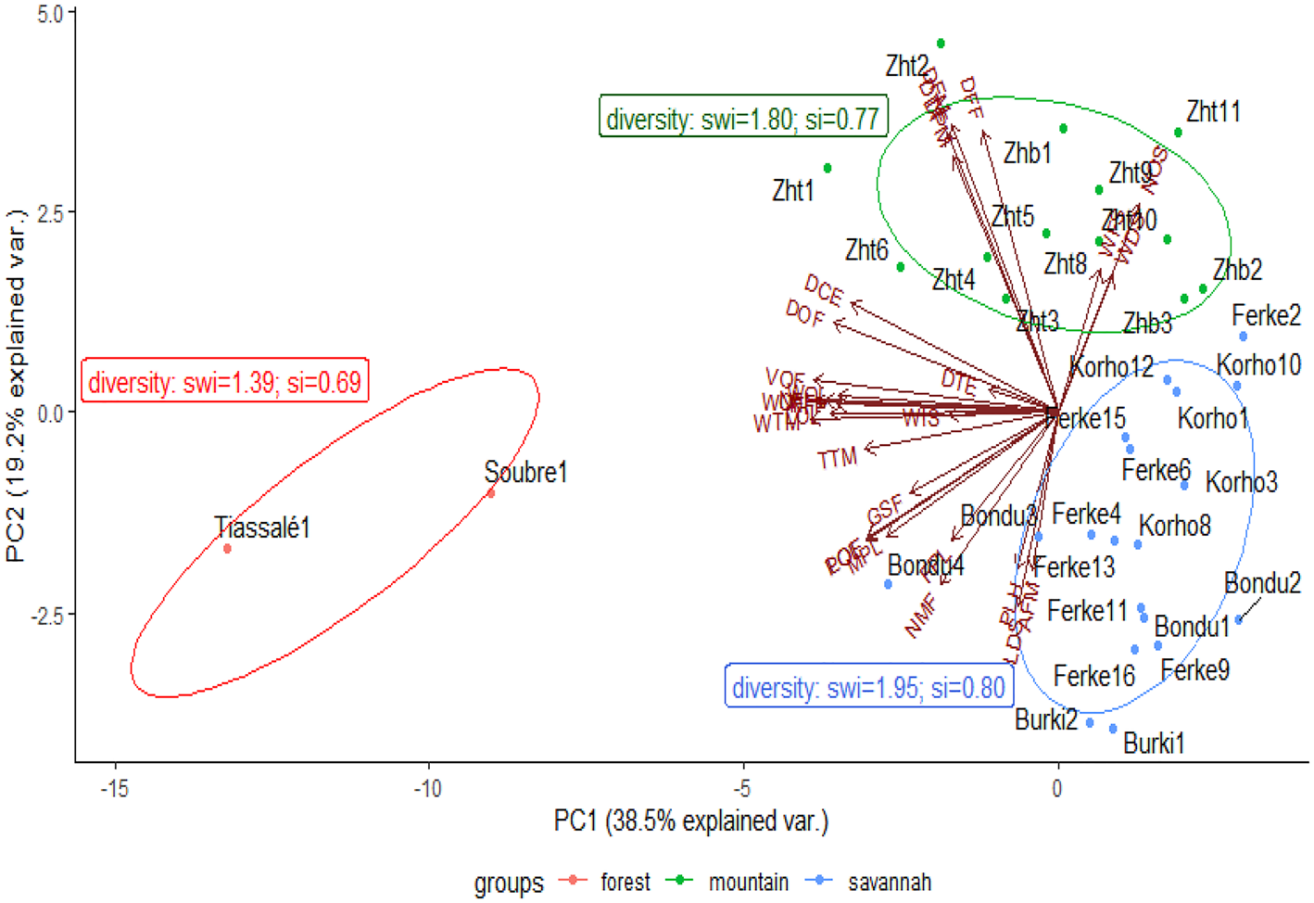
Separation of the morphotypes from the forest, mountain, and savannah regions and vectors of morphological and phenological traits obtained from the principal components analysis along with the diversity indices in each region.

A principal components analysis (Fig. 3) separated the morphotypes in distinct clusters. The morphotypes from the forest region form a distant cluster in the lower left of the two-dimensional representation of the first two principal components. The morphotypes of the mountain region form another cluster at the upper-right of the biplot. And the morphotypes from the savannah are grouped at the lower-right. The vectors indicate the traits that most characterize the morphotypes of a given region. It appears that the length of the vector is an indication of the degree of significance of the trait in the differentiation of the accessions. There is no evidence that the characters number of day to 50% emergence, weight of dry seeds and weight of fresh seeds exerted any genotypic differentiation. However, girth size and all fruit-related traits such as number of fruits per plant, diameter of the fruit, thickness of the pulp, weight of the fruit, diameter of the cavity enclosing the seeds, and volume of the fruit set the morphotypes of the forest region apart. The number of seeds per fruit and the phenological traits including the number of days from seeding to first female and male flower appearances, number of days to 50% flowering and number of days to physiological maturity are strong characteristics of the morphotypes from the mountain region. The morphotypes of the savannah region diverged morphologically with longer plant height, and accelerated phenology with shorter vegetative and reproductive phases. The morphological and phenological divergence of the accessions from the three regions is reflective of the ecosystems where they are thriving, to the point that the accessions from a region may be considered a separate variety or ecotype. All three regions showed high diversity of *C. moschata* with a Shanon-Weaver diversity index ranging between 1.39 for the forest region, to 1.95 for the savannah region. The Shanon-Weaver index is an indicator of the richness in term of number of different genotypes of *C. moschata* in a region, and evenness meaning that the different genotypes are represented in fairly equal proportion^43–45^. The Simpson index is an indicator of evenness. It scales between 0 and 1. The Simpson indices are 0.69 for the forest region, 0.77 for the mountain region and 0.80 for the savannah region (Fig. 3). The computation of the two indices takes into account the sample size. And the lower Shannon–Weaver and Simpson indices for the forest region could be due to smaller sample size compared to the other regions.

## Discussion

*Cucurbita moschata* has migrated to Cote d’Ivoire from Latin America with the intercontinental maritime exchanges that began in the fifteenth century. *C. moschata* has become an integral part of the Cote d’Ivoire’s landscape where it adapted to different ecosystems. A phenological and morphological analysis of *C. moschata* shows large variability for all assessed traits between the sampling habitats where the crop is largely grown. Further analysis of the structure of the population of *C. moschata* in Cote d’Ivoire indicates the existence of three clines, or ecotypes, that thrive in three separate and distinct ecosystems with their respective bioclimatic parameters. The forest region clines are comparatively shorter in height with larger girth size, larger dimensions of the leaves and carry about 2.7 fruits per plant. They have remarkably large fruits that are heavier with a thick pulp and a large diameter of the cavity containing the seeds. They comparatively have smaller number but large seeds. And they form a distant cluster with a very high genotypic diversity indicated by a high median dissimilarity rank and outliers. The savannah clines are longer in height with small girth size and smaller dimensions of the leaves. They carry about 1.34 fruits per plant and the fruits are smaller. They reach physiological maturity faster and have a high growth rate. The mountain clines are characterized by a delayed phenology with longer periods from seeding to flowering and to physiological maturity. They carry about 1.49 fruits per plant and the plants are short. The fruits have a larger number of seeds, larger weights of fresh and dry seeds, but the seeds are smaller compared to the size of the seeds from the forest region clines.

The observed genotypic differentiation of the clines is likely due to different mechanisms of adaptation that evolved through natural selection in order to better adapt and thrive in the different ecosystems with their bioclimatic exigencies and constraints. In the natural habitat, organisms must make changes to accommodate their physiology and anatomy to utilize the available resources of their environment. For example, an examination of the wood frog in the tundra of Canada, the mountains of Virginia and the lowlands of Maryland showed that observed larval developmental patterns were locally adaptive and reflected differential selection pressures unique to each environment. Environmental differences (especially temperature) accounted for most of the observed phenotypic variation^46^. In the current study, the three ecotypes adopted different mechanisms of adaptation to their respective environments. The mountain region characterized by a very long rainy season and a short dry season, with a temperature of 27 °C on the average and a relative humidity of 69% likely favors a longer retention of soil moisture and the availability of water to the growing plant. Genotypes of *C. moschata* in that region expressed delayed phenology with longer period of the vegetative phase and longer period to reach physiological maturity. They have a relatively higher seed yield. On the other hand, the bioclimatic parameters of the savannah region are characterized by a short rainy season and a long dry season, higher mean temperature and higher relative humidity that contribute to a shorter period of soil moisture retention and water availability to the growing plant. Accessions of the savannah region have accelerated phenology and a high growth rate to accommodate the short rainy season. They have longer plant height with small girth size and small leaves likely to reduce loss of water through transpiration under the high humidity and high temperature, while fulfilling their ontogenetic needs to produce harvestable fruits. Compared to the other two environments, the forest region appears to present bioclimatic parameters favorable for the growth of accessions of *C. moschata*. The forest region has two rainy seasons that alternate with two dry seasons and contribute to a better soil moisture retention and availability of appropriate quantity of water for the growing plant. With an average temperature of 27 °C and a relative humidity of 70%, it presents the best environment for the growth of the accessions of *C. moschata* in Cote d’Ivoire with larger leaves, longer peduncles, longer petioles, larger girth size, comparatively higher fruit yield, larger volume of the fruit and thicker fruit pulp. In an environment with low water availability, high water-use efficiency correlates with small leaf size and small organs, while larger organs are the norm in an environment with high water availability^47^. Smaller organ sizes may be favored in drier habitats because, for example, smaller leaves provide less surface area for transpiration water loss and smaller organ and plant size can reduce developmental time^6^.

Differences in the size of the organs may be due to differences in cell numbers and/or cell sizes. For example, divergence in the body size of *Drosophilla melanogaster* from two geographically isolated regions are linked to cytological differences, one caused by a variation in cell number and the other one by both cell number and cell size, attributable to diverse genetic mechanisms^48^. In this study, the three clines of *C. moschata* evolved as the result of local adaptation through natural selection in order to thrive in the ecologically different environments. The clines differ mostly in sizes of organs and phenology as responses to what each environment can allow for the full completion of the ontogeny of the accessions. Obviously, anatomical and physiological differences in response to the environments resulted in the observed differences in sizes and weights of organs, and phenology of the accessions of *C. moschata*. To meet local environmental conditions, accessions of *C. moschata* developed different genetic mechanisms which underlie the observed genotypic divergence. Clearly, the accessions are pre-disposed to survive and adapt to the new ecosystems in order to do so and to persist. Pre-existing variation for plasticity permitted the accessions to persist under the new environments and over time, the persistence allowed new genetic variation to arise through mutations and/or recombination^3,8^ that led to the adaptive divergence of the accessions and the emergence of the ecotypes of *C. moschata*.

## Conclusion

*Cucurbita moschata* is a native crop of Latin America that spreads to Africa, Asia and Europe with its predisposition for adaptation to various ecosystems. In Cote d’Ivoire, *C. moschata* grows in several habitats and expresses large morphological and phenological variability along with outliers. Three clines of *C. moschata* are distinguished and their developmental needs, their morphology and their phenology are in congruence with the three distinct ecosystems and their respective bioclimatic parameters. The allopatric formation of the three clines is likely conditioned by soil water availability for the ontogeny of the plant.

## Material and methods

### Origin of germplasm

**Figure 4:**
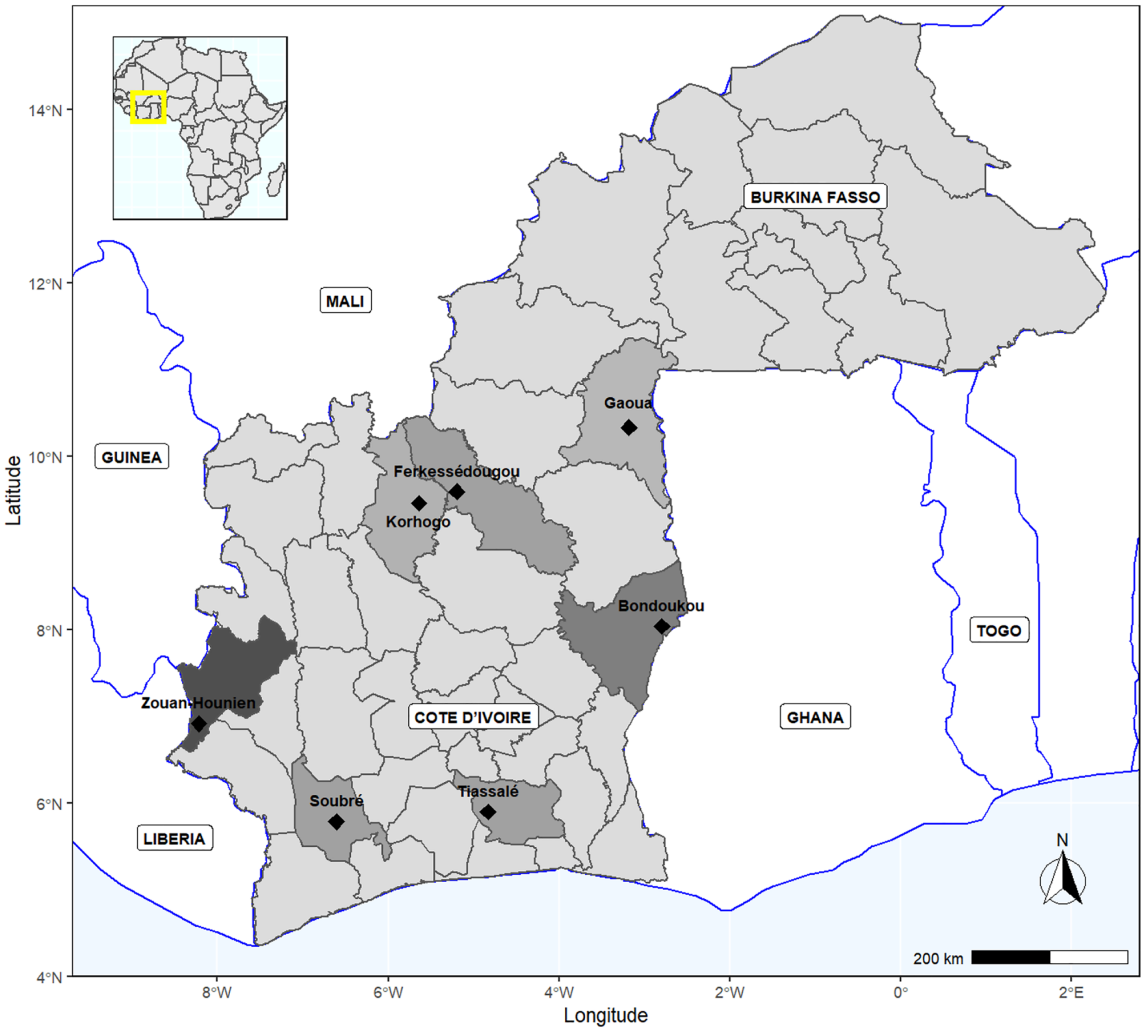
Origin of the accessions of *Cucurbita moschata* in Cote d’Ivoire and Burkina Fasso (map produced with R, version 4.2.1; available online https://cran.r-project.org).

The plant material consists of 85 accessions from 34 morphotypes of *Cucurbita moschata* maintained in the germplasm bank of University Nangui Abrogoua. They are collected from six sites in Cote d’Ivoire and one site in Burkina Fasso (Fig. 4). The distribution of the accessions used in this study is as follows: Twenty-one accessions are from the North of Cote d’Ivoire, including 11 accessions from the region of Korhogo (Korho) and 10 accessions from the region of Ferkessedougou (Ferke). Twenty-five accessions are from the West of Cote d’Ivoire, in the region of Zouan-Hounien (Zh). Nineteen accessions are from the North-East of Cote d’Ivoire in the region of Bondoukou (Bondu), 5 accessions are from the South-West in the region of Soubre and 5 accessions are from the South of Cote d’Ivoire, in the region of Tiassale. Finally, 10 accessions are from the South-West of Burkina Faso, in the region of Gaoua (Burki). The geographic coordinates of the collection sites of the accessions span from 5°47’ N to 10°21’ N and from 8°13’ W to 2°48’W (Fig. 4). Table 4 gives a detail on the origin, number of accessions, geographic coordinates, weather parameters and types of vegetation of the collection sites of the accessions of *Cucurbita moschata* germplasm.

### Experimental methods

**Figure 5:**
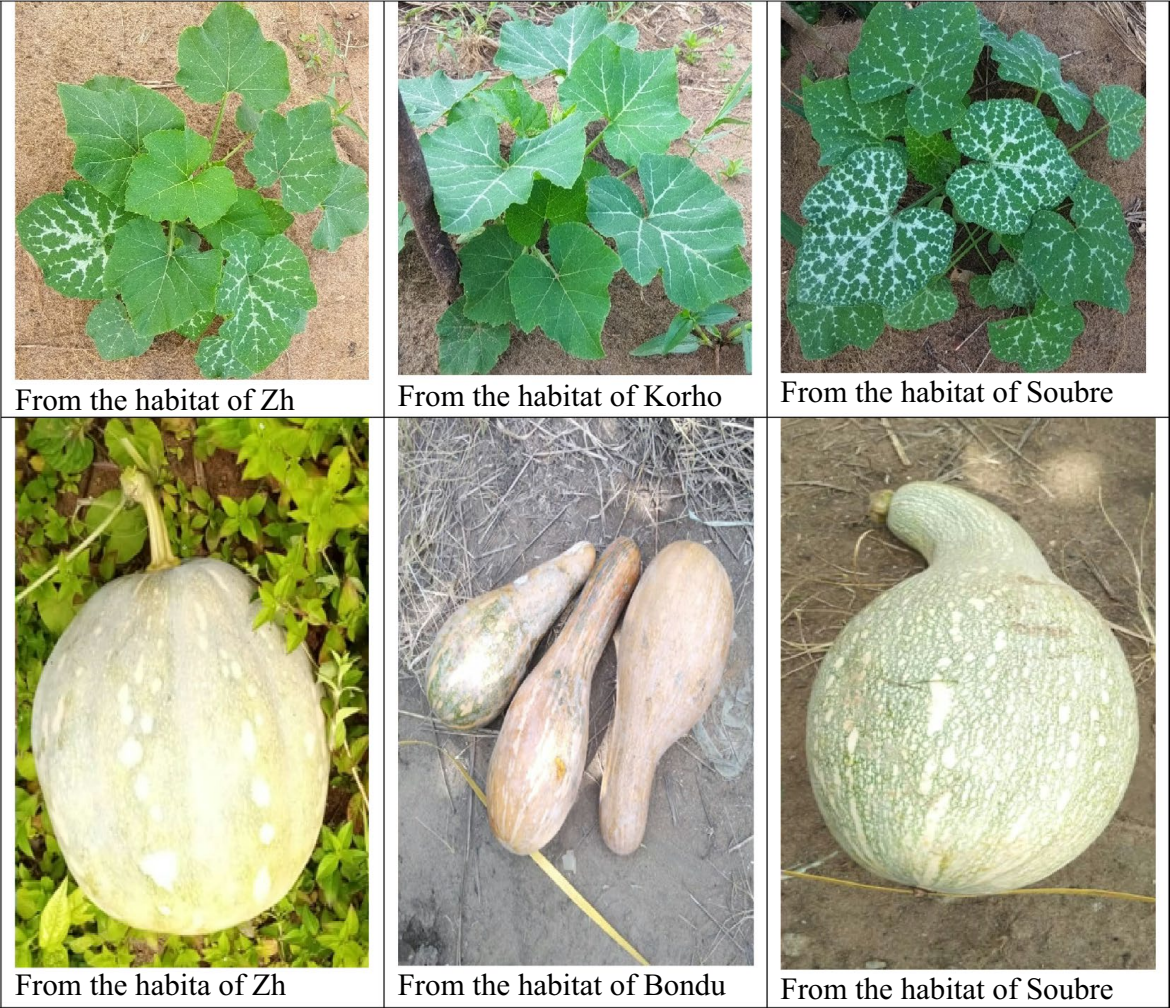
Seedlings and fruits of *C. moschata* from some of the sampled habitats.

The experiment was conducted at the experimental station of University Nangui Abrogoua, Abidjan (4° 10′ 20’’ W, 5° 35′ 20’’ N), Cote d’Ivoire, in 2019 and 2020. The climate of Abidjan is characterized by two rainy seasons from April to July and October to November and two dry seasons from December to March and August to September. But, this pattern of alternating seasons has been disturbed in recent years. The annual rainfall in Abidjan is over 1200 mm, the daily mean temperature is 27 °C, and the average relative humidity is 70%. The vegetation is characterized by a dense forest and the ferralitic soil is rich in organic matter. The seeding date for the first year of the experiment was September 10th, 2019. Due to changing weather patterns, the usually short rainy season started in early September of that year and sparsely extended to December. The seeding date for the second year of the experiment was April 20th, 2020, at the onset of the long rainy season. The experiment was arranged in a randomized complete block design with three replications and covered a total area of 0.816 ha in both 2019 and 2020. The accessions were randomly assigned to different plots in each block and the blocks were randomized. Each plot occupied an area of 288 m^2^ (12 m × 24 m) and was composed of 5 rows. The space between rows, and between plants within a row was 3 m, resulting in a planting density of 1190 plants ha^−1^. Each year a total, 335 plants were randomly sampled, followed from emergence to physiological maturity, and used for all observations and measurements. Figure 5 presents some images of the seedlings and the fruits of the accessions of *C. moschata* of the current study. All agricultural practices were followed according to recommendations.

### Assessment of phenological, agro-morphological and yield traits

The assessed agro-morphological characteristics are reported in Table 5. We followed the morphological descriptors suggested by Bioversity International and the European Cooperative Programme for Plant Genetic Resources.

**Table 5:** Phenological, vegetative and flowering and yield traits of *C. moschata* assessed in this study, along with their descriptions and unit of measure in parenthesis.

Abbreviated name of traits	Description and (unit of measure)
Phenological	
DTE	Days to 50% emergence (d). Number of days from seeding to 50% emergence in a plot
DTF	Days to 50% flowering in a plot (d)
DFM	Days to first male flower appearance in a plot (d)
DFF	Days to first female flower appearance in a plot (d)
DPM	Days to physiological maturity in a plot (d)
AFM	Days from first female flower appearance to fruit maturity in a plot (d)
Vegetative	
PLH	Plant height (cm)
GSF	Girth size at anthesis (cm)
LOL	Leaf length (cm)
WOL	Leaf width (cm)
MPL	Peduncle length of male flower (cm)
FPL	Peduncle length of female flower (cm)
LOP	Petiole length (cm)
Flowering and yield	
NMF	Number of male flowers per plant (unit)
NFF	Number of female flowers per plant (unit)
NFP	Number of fruits per plant (unit)
LOF	Length of fruit (cm)
DOF	Fruit diameter (cm)
VOF	Volume of fruit (cm^3^). Estimated with the formula VOF = $(\pi *{\text{DOF}}^{{3}} {)/6}$
WOF	Weight of fruit (g)
DCE	Diameter of the cavity enclosing the seeds (cm)
TTM	Thickness of the pulp (cm)
WTM	Weight of the pulp (g)
NOS	Number of seeds per fruit (unit)
WFS	Weight of the fresh seeds per fruit (g)
WDS	Weight of the dry seeds per fruit (g). Obtained after placing the fresh seeds in an oven for 3 days at 70 °C
LDS	Length of a dry seed (mm). Average of longest axis of 30 seeds
WIS	Width of a dry seed (mm). Average of second longest axis of 30 seeds

### Statistical model and analysis

The factors, block, accessions and year were considered random and we fitted a random effects model to get a better assessment of the components of variance. We used the following model:

$$Y_{ijk} = \mu + \beta_{i} + \alpha_{j} + \tau_{k} + \varepsilon_{ijk}$$

where $$\mu$$ is the overall mean, $$\beta_{i}$$ is the random effect of block, $$\alpha_{j}$$ is the random effect of year, $$\tau_{k}$$ is the random effect of accession (genotype) and $$\varepsilon_{ijk}$$ is the random error term. The model assumes that $$\beta_{i} {, }\alpha_{{j{,}}} {\text{ and }}\tau_{k}$$ are independently distributed with Normal distributions having a mean zero and respective variances $$\sigma_{\beta }^{2} ,$$ $$\sigma_{\alpha }^{2} ,$$ and $$\sigma_{\tau }^{2}$$. The analysis showed that the differences between blocks were not significant and the model was reduced with the removal of the random effect of block. In addition, for many response variables, the year to year difference was null and for those traits the model was further reduced to only retain the main treatment variable,$$\tau_{k}$$. With the *lme4* package^37^ of the R statistical software^39^, we obtained the genotypic variance ($$\sigma_{g}^{2} )$$ which is $$\sigma_{\tau }^{2}$$, the residual variance ($$\sigma_{e}^{2} )$$ and, when possible, the year to year variance ($$\sigma_{\alpha }^{2}$$). The phenotypic variance is $$\sigma_{p}^{2} =$$$$\sigma_{g}^{2}$$ + $$\sigma_{e}^{2}$$ and the quantitative genetic differentiation^6,38,49^ for a trait between accessions across all habitats is given by $$Q_{ST} = \sigma_{g}^{2} /(\sigma_{g}^{2} + 2\sigma_{e}^{2} )$$. Phenotypic coefficient of variation (*CVp*), genotypic coefficient of variation (*CVg*) and environmental coefficient of variation (*CVe*) are respectively computed as follow: $$CV_{p} = {{\left( {100*\sqrt {\sigma_{p}^{2} } } \right)} \mathord{\left/ {\vphantom {{\left( {100*\sqrt {\sigma_{p}^{2} } } \right)} \mu }} \right. \kern-0pt} \mu }$$, $$CV_{g} = {{\left( {100*\sqrt {\sigma_{g}^{2} } } \right)} \mathord{\left/ {\vphantom {{\left( {100*\sqrt {\sigma_{g}^{2} } } \right)} \mu }} \right. \kern-0pt} \mu }$$ and $$CV_{e} = {{\left( {100*\sqrt {\sigma_{e}^{2} } } \right)} \mathord{\left/ {\vphantom {{\left( {100*\sqrt {\sigma_{e}^{2} } } \right)} \mu }} \right. \kern-0pt} \mu }$$. The *tidyverse* package^50^ was used for data management. We computed the mean effect of each trait per sampling habitat, and used the Fisher’s least significant difference (LSD) procedure to separate means that are significantly different.

### Clustering accessions and identification of ecotypes

The segmentation procedure used to regroup closely related accessions is the hierarchical agglomerative clustering. With this procedure, each accession is assigned to its own cluster. The distances between clusters are computed and the clusters with the shortest distance are merged to form a new cluster, as they are the closest to each other. The distances are then recomputed and the process is repeated until all accessions are regrouped in one cluster. We used the *hclust* function in the *stat* package of R, version 4.2.1^39^ with the “ward.D2” method^51^. We then used the *phylo* function of the *ape* package^41^ for visualization of the structure of the populations of *C. moschata* in Cote d’Ivoire. The *NbClust* package^40^ helped to determine the number of clusters, based on the majority rule. For the purpose of clarity, we used the averages of the accessions of the 34 morphotypes. We then conducted a principal components analysis (pca). The pca is a technique for reducing the dimensionality of a large data set while preserving most of its variability. It does so by creating new uncorrelated variables called principal components and concentrates the maximum variance in the first few components^52^. The technique increases interpretability and allow low-dimensional graphical representation of the data set. With those advantages of the pca, we graphically presented the accessions along with the variable vectors in the two axes formed by the first two principal components that have the largest variances. This graphical representation helped to assess the morphological traits that best characterize the accessions grouped together according to their similarities in the attempt to identify the ecotypes of *C. moschata* in Cote d’Ivoire, along with their particularities. We used the *ggbiplot* package^53^ for the visualization of the biplot.

### Compliance to plant material collection guidelines

For the sampling of the accessions of Cucurbita moschata, all procedures were conducted in accordance with the guidelines. And permission was given to collect the accessions from the sampling sites of this study.

## Supplementary Information

Supplementary Information.

Supplementary Table S1.

Supplementary Table S2.

## Data Availability

All data generated or analyzed during this study are included in this published article as part of the Supplementary Information.

## References

[1] Gomulkiewicz R, Holt RD (1995). When does evolution by natural selection prevent extinction?. Evolution.

[2] Pease CM, Lande R, Bull JJ (1989). A model of population growth, dispersal and evolution in a changing environment. Ecology.

[3] Ackerly DD (2000). The evolution of plant ecophysiological traits: Recent advances and future directions. Bioscience.

[4] Matesanz S, Horgan-Kobelski T, Sultan SE (2012). Phenotypic plasticity and population differentiation in an ongoing species invasion. PLoS ONE.

[5] Gentili R (2018). Genetic differentiation, local adaptation and phenotypic plasticity in fragmented populations of a rare forest herb. PeerJ.

[6] Gao S-b, Mo L-d, Zhang L-h, Zhang J-l, Wu J-b, Wang J-l, Zhao N-x, Gao Y-b (2018). Phenotypic plasticity vs. local adaptation in quantitative traits differences of Stipa grandis in semiarid steppe, China. Nat. Sci. Rep..

[7] Bürger R, Lynch M (1995). Evolution and extinction in a changing environment: A quantitative-genetic analysis. Evol..

[8] Pigliucci M (2005). Evolution of phenotypic plasticity: Where are we going now?. Trends Ecol Evol..

[9] Darwin C (1859). On the Origin of Species by Means of Natural Selection, or the Preservation of Favoured Races in the Struggle for Life.

[10] Gregory TR (2009). Understanding natural selection: Essential concepts and common misconceptions. Evol. Edu. Outreach.

[11] Bradshaw A.D. (1984). Adaptation of Plants to Soils Containing Toxic Metals – A Test for Conceit (Pages: 4–19). In Ciba Foundation Symposium 102 - Origins and Development of Adaptation, Volume 102. ISBN:9780470664209. DOI:10.1002/9780470720837. 10.1002/9780470720837.ch2 6559118

[12] Memon AR, Aktoprakligil D, Özdemir A, Vertii A (2001). Heavy metal accumulation and detoxification mechanisms in plants. Turk. J. Bot..

[13] Gwanama C, Labuschagne MT, Botha AM (2000). Analysis of genetic variation in *Cucurbita moschata* by random amplified polymorphic (RAPD) markers. Euphytica.

[14] Ferriol M, Picó B, Fernández P, Nuez F (2004). Molecular diversity of a germplasm collection of squash (*Cucurbita moschata*) determined by SRAP and AFLP markers. Crop Sci..

[15] Aruah CB, Uguru MI, Oyiga BC (2010). Variations among some Nigerian *Cucurbita* landraces. Afr. J. Plant Sci..

[16] Barboza N, Albertazzia FJ, Sibaja-Corderob JA, F. Mora-Uma˜nac, C. Astorgad, P. Ramírez (2012). Analysis of genetic diversity of Cucurbita moschata (D.) germplasm accessions from Mesoamerica revealed by PCR SSCP and chloroplast sequence data. Scientia Horticulturae.

[17] Darrudi R, Nazeri V, Soltani F, Shokrpour M, Ercolano MR (2018). Genetic diversity of Cucurbita pepo L. and Cucurbita moschata Duchesne accessions using fruit and seed quantitative traits. J. Appl. Res. Med. Aromat. Plants.

[18] Ezin V, Gbemenou UH, Ahanchede A (2022). Characterization of cultivated pumpkin (Cucurbita moschata Duchesne) landraces for genotypic variance, heritability and agro-morphological traits. Saudi J. Biol. Sci..

[19] Gbemenou UH, Ezin V, Ahanchede A (2022). Current state of knowledge on the potential and production of *Cucurbita moschata* (pumpkin) in Africa: A review. Afr. J. Plant Sci..

[20] Santa-Cruz JH, Kump KL, Arellano C, Goodman MM, Krakowsky MD, Holland JB, Balint-Kurti PJ (2014). Yield effects of two southern blight resistance loci in maize hybrids. Crop Sci..

[21] Dong OX, Ronald PC (2019). Genetic engineering for disease resistance in plants: Recent progress and future perspectives. Plant Physiol..

[22] Rana JC, Sharma TR, Tyagi RK, Chahota RK, Gautam N, Singh M (2015). Characterisation of 4274 accessions of common bean (Phaseolus vulgaris L.) germplasm conserved in the Indian gene bank for phenological, morphological and agricultural traits. Euphytica.

[23] Gomes RS, Almeida CF, Costa JRS, Machado Junior R, Delazzari F, Silva FCS (2016). Genetic diversity in sweet cassava from the Brazilian Middle North Region and selection of genotypes based on morphoagronomical descriptors. Afr. J. Agr. Res..

[24] Lee H-Y, Jang S, Yu C-R, Kang B-C, Chin J-H, Song K (2021). Population structure and genetic diversity of cucurbita moschata based on genome-wide high-quality SNPs. Plants.

[25] Whitaker TW, Davis GN (1962). *Cucurbits*: Botany, cultivation and utilization. New York.

[26] Gomes RS, Machado Junior R, de Almeida CF, Chagas RR, de Oliveira RL, Delazari FT (2020). Brazilian germplasm of winter squash (*Cucurbita moschata* D.) displays vast genetic variability, allowing identification of promising genotypes for agro-morphological traits. PLoS ONE.

[27] Hancock, J. F. Plant evolution and the origin of crop species / James F. Hancock. 2nd ed. (2004). Library of Congress Cataloging-in-Publication Data. CABI Publishing. ISBN 0 85199 685 X.

[28] Robinson RW, Decker-Walters DS (1997). Cucurbits.

[29] Piperno DR, Andres T, Stothert KE (2000). Phytoliths in cucurbita and other neotropical cucurbitaceae and their occurrence in early archaeological sites from the lowland American tropics. J. Archaeol. Sci..

[30] Lira R, Andres TC, Nee M, Lira R (1995). Cucurbita L. Estudios Taxonómicos y Ecogeográficos de las Cucurbitaceae Latinoamericanas de Importancia Económica:Cucurbita, Sechium, Sicana y Cyclanthera, Systematic and Ecogeographic Studies on Crop Genepools.

[31] Filov AI (1966). Ekologija i klassifikatzija tykuy. Bjulleten Glavnogo Botaniceskogo Sada.

[32] Youn SJ, Chung HD (1998). Genetic relationship among the local varieties of Korean native squashes (Cucurbita moschata) using RAPD technique. J. Kor. Soc. Hortic. Sci..

[33] Baranek M, Stift G, Vollmann J, Lelley T (2000). Genetic diversity within and between the species Cucurbita pepo, C. moschata and C. maxima as revealed by RAPD markers. Cucurbit Genet. Coop. Rep..

[34] Men X, Choi SI, Han X, Kwon H-Y, Jang G-W, Choi Y-E, Park S-M, Lee O-H (2021). Physicochemical, nutritional and functional properties of Cucurbita moschata. Food Sci. Biotechnol..

[35] OECD. (2012). Consensus Document on the Biology of *Cucurbita* L. (Squashes, Pumpkins, Zucchinis and Gourds). OECD Environment, Health and Safety Publications. Series on Harmonization of Regulatory Oversight in Biotechnology. No. 53. Paris, France.

[36] Esquinas-Alcazar J.T. and Gulick P.J. (1983). Genetic resources of Cucurbitaceae: A global report. IBPGR.

[37] Bates B, Maechler M, Bolker B, Walker S (2015). Fitting linear mixed-effects models using lme4. J. Stat. Softw..

[38] Spitze K (1993). Population structure in Daphnia obtusa: Quantitative genetic and allozymic variation. Genetics.

[39] R Core Team (2022). R: A language and environment for statistical computing. R Foundation for Statistical Computing, Vienna, Austria. https://www.R-project.org/.

[40] Charrad M, Ghazzali N, Boiteau V, Niknafs A (2014). NbClust: An R Package for Determining the Relevant Number of Clusters in a Data Set. J. Stat. Softw..

[41] Paradis E, Schliep K (2019). ape 5.0: An environment for modern phylogenetics and evolutionary analyses in R. Bioinformatics.

[42] Hennig C. fpc: Flexible Procedures for Clustering. R package version 2.2–9, https://CRAN.R-project.org/package=fpc (2020).

[43] Kindt R, Coe R (2005). Tree Diversity Analysis: A Manual and Software for Common Statistical Methods for Ecological and Biodiversity Studies.

[44] Magurran AE (1988). Ecological Diversity and its Measurement.

[45] Rousseau D, Van Hecke P, Nijssen D, Bogaert J (1999). The relationship between diversity profiles, evenness and species richness based on partial ordering. Environ. Ecol. Stat..

[46] Berven KA, Gill DE (1983). Interpreting geographic variation in life-history traits. Am. Zool..

[47] Dudley SA (1996). Differing selection on plant physiological traits in response to environmental water availability: A test of adaptive hypotheses. Evolution.

[48] Gilchrist AS, Partridge L (1999). A comparison of the genetic basis of wing size divergence in three parallel body size clines of *Drosophila melanogaster*. Genetics.

[49] Merilä J, Crnokrak P (2001). Comparison of genetic differentiation at marker loci and quantitative traits. J. Evol. Biol..

[50] Wickham H, Averick M, Bryan J, Chang W, McGowan LD, François R, Grolemund G, Hayes A, Henry L, Hester J, Kuhn M, Pedersen TL, Miller E, Bache SM, Müller K, Ooms J, Robinson D, Seidel DP, Spinu V, Takahashi K, Vaughan D, Wilke C, Woo K, Yutani H (2019). Welcome to the tidyverse. J. Open Source Softw..

[51] Murtagh F, Legendre P (2014). Ward's hierarchical agglomerative clustering method: Which algorithms implement Ward’s criterion?. J. Classif..

[52] Jolliffe IT, Cadima J (2016). Principal component analysis: A review and recent developments. Phil. Trans. R. Soc. A.

[53] Vu VQ _ggbiplot: A ggplot2 based biplot_. R package version 0.55, http://github.com/vqv/ggbiplot (2011).

