# Supplementary material for: Assessment of the variability of the morphological traits and differentiation of Cucurbita moschata in Cote d’Ivoire
Source: Sci Rep. 2023 Mar 6;13:3689. doi: 10.1038/s41598-023-30295-7 (PMC9988981; doi:10.1038/s41598-023-30295-7)
Supplement: Supplementary file 1 — Supplementary Information. [file 41598_2023_30295_MOESM1_ESM.docx]

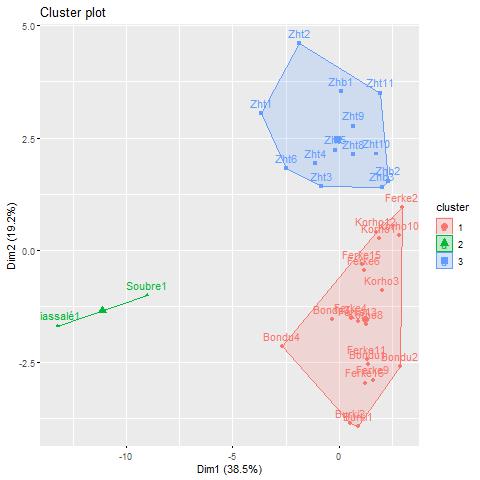


Supplemsssentary material: Clustering with the K-means algorithm. Accessions forming a cluster are from the same geographical zone.
